# Key biomarkers in type 2 diabetes patients: A systematic review

**DOI:** 10.1111/dom.15991

**Published:** 2024-10-02

**Authors:** Thien Ngoc Le, Richard Bright, Vi‐Khanh Truong, Jordan Li, Rajiv Juneja, Krasimir Vasilev

**Affiliations:** ^1^ College of Medicine and Public Health Flinders University Adelaide South Australia Australia; ^2^ Department of Renal Medicine, Flinders Medical Centre Bedford Park South Australia Australia

**Keywords:** biomarkers, cytokines, glucose intolerance, glycaemic index, hyperglycaemia, inflammation, lipids, T2DM, type 2 diabetes

## Abstract

Type 2 diabetes mellitus (T2DM) is not just a local health issue but a significant global health burden, affecting patient outcomes and clinical management worldwide. Despite the wealth of studies reporting T2DM biomarkers, there is an urgent need for a comparative review. This review aims to provide a comprehensive analysis based on the reported T2DM biomarkers and how these are linked with other conditions, such as inflammation and wound healing. A comparative review was conducted on 24 001 study participants, including 10 024 T2DM patients and 13 977 controls (CTL; age 30–90 years). Four main profiles were extracted and analysed from the clinical reports over the past 11 years: haematological (1084 cases vs. 1458 CTL), protein (6753 cases vs. 9613 CTL), cytokine (975 cases vs. 1350 CTL) and lipid (1212 cases vs. 1556 CTL). This review provides a detailed analysis of the haematological profile in T2DM patients, highlighting fundamental changes such as increased white blood cells and platelet counts, accompanied by decreases in red blood cell counts and iron absorption. In the serum protein profile, a reduction in albumin and anti‐inflammatory cytokines was noted along with an increase in globulin levels and pro‐inflammatory cytokines. Furthermore, changes in lipid profiles were discussed, specifically the decreases in high‐density lipoprotein (HDL) and the increases in low‐density lipoprotein (LDL) and triglycerides. Understanding the changes in these four biomarker profiles is essential for developing innovative strategies to create diagnostic and prognostic tools for diabetes management.

## 1 INTRODUCTION

**FIGURE 1:**
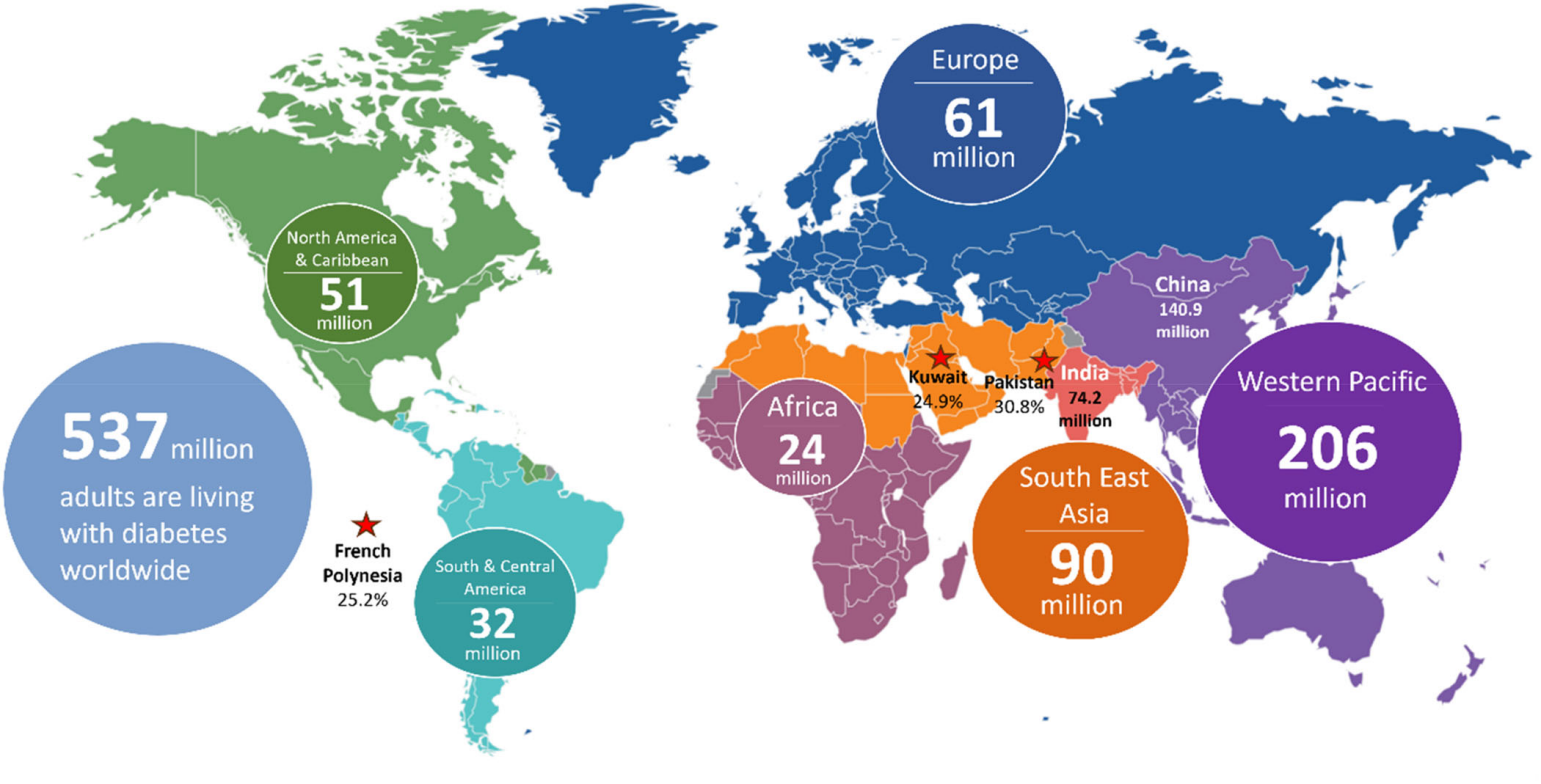
Global diabetes demographics in 2021. Diabetes has the most significant impact in the Western Pacific Region (WPR), where 206 million adults are affected. Data were sourced from the IDF Diabetes Atlas 2021.^3^

The term ‘diabetes mellitus’ was introduced in 1674 by Thomas Willis.^1^ According to the World Health Organization (WHO), diabetes is the epidemic of the 21st century.^2^ The International Diabetes Federation (IDF) estimated that 537 million adults aged 20–79 years are living with diabetes worldwide (Figure 1).^3^ There are three main types of diabetes mellitus: type 1, type 2 diabetes mellitus (T2DM) and gestational diabetes. T2DM accounts for 90%–95% of all diabetes cases.^2^ T1DM is recognized as an autoimmune disease. It occurs due to the destruction of β cells, which are responsible for producing insulin in the pancreatic islets by T cells, resulting in a high blood glucose concentration. T2DM is a chronic disease characterized by two pathophysiological mechanisms: (1) decrease (20%–65%) in the mass and quantity of pancreatic β cells, leading to impaired insulin secretion^4^; (2) when insulin becomes less effective, cells in muscles, fat and liver fail to respond adequately to insulin. As a result, more insulin is required to control blood glucose concentration by inhibiting glucose production in the liver and inducing glucose adsorption in muscle and adipose cells. Over time, elevated levels of circulating insulin cause insulin resistance in the body, and the pancreas does not produce enough insulin to overcome the cells' resistance. T2DM is a complex disease influenced by a combination of genetic, environmental and lifestyle factors.

According to the Diabetes Atlas 2021, as shown in Figure 1, the Western Pacific Region is the most affected by diabetes, with 206 million adults living with the condition. This high prevalence is driven by factors such as ageing populations, urbanization, increased obesity and genetic predispositions. The region faces significant public health and economic challenges due to the growing diabetes epidemic, emphasizing the need for effective prevention and management strategies. The regions predominantly affected by the disease are China and India, with approximately 140.9 million and 74.2 million adults affected, respectively. The top three countries with the highest rate of diabetes prevalence in adults (20–79 years) are Pakistan (30.8%), French Polynesia (25.2%) and Kuwait (24.9%).^5^ The most significant impact of diabetes depends not only on its prevalence but also on factors such as access to and availability of diagnostic facilities and treatment options along with sociodemographic, cultural and health system influences. A significant association between changes in plasma protein composition and concentrations and increased risk of T2DM has been demonstrated (Table 2). This review focuses on four key biomarker profiles – haematological, protein, cytokine and lipid – in early‐stage T2DM patients due to their significant roles in the disease's pathophysiology. Haematological changes can indicate inflammation and complications; protein levels can reflect metabolic processes and damage; cytokine imbalances show inflammation and insulin resistance, and lipid abnormalities highlight cardiovascular risks. Examining these interconnected profiles, the review aims to provide a comprehensive understanding of how these biomarkers impact inflammatory conditions and wound healing in T2DM and identify potential biomarkers. Changes in glucose metabolism and immune response can begin to affect wound healing, even if the disease is not yet advanced.^15^ Understanding how these biomarkers influence inflammation and wound healing can lead to the development of targeted therapies that modulate these biological processes.

**FIGURE 2:**
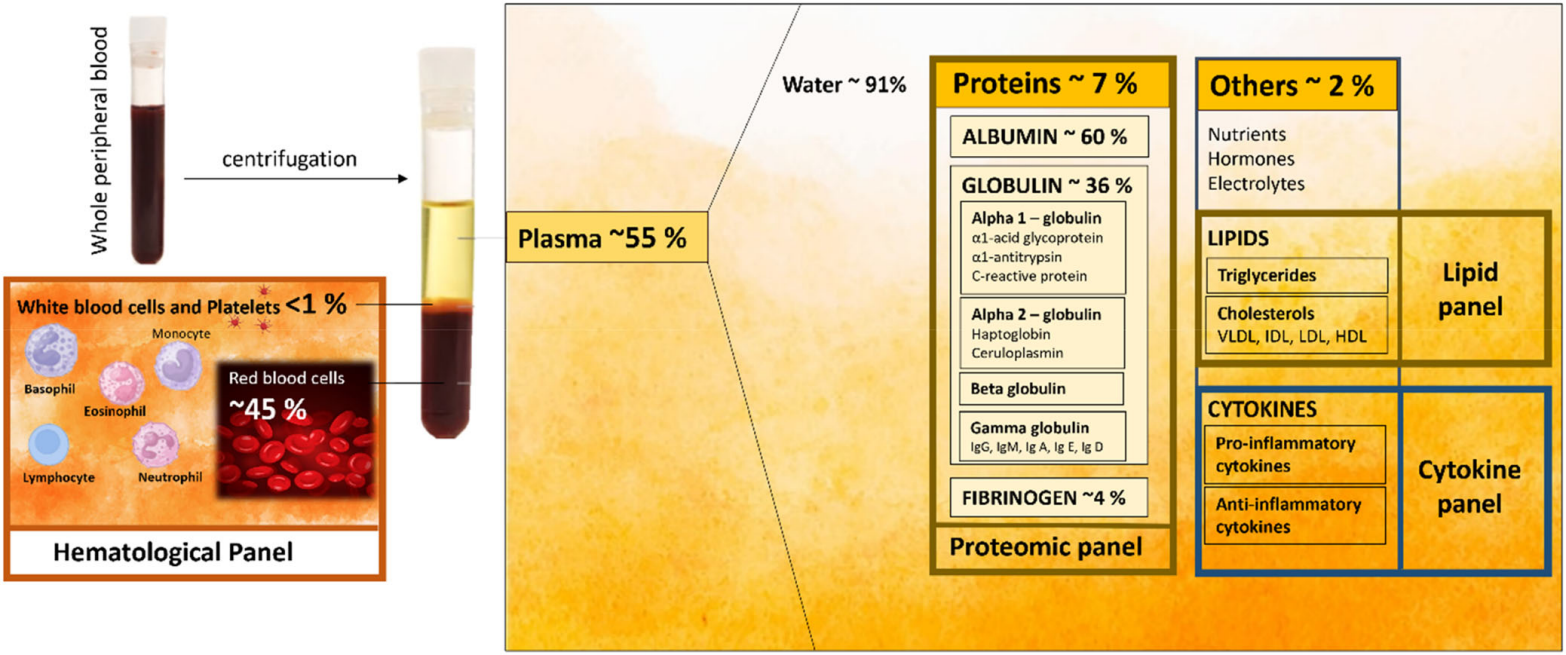
Biomarkers for type 2 diabetes mellitus (T2DM) include four profiles: haematological, proteomic, lipid and cytokine.

In 2021, 1.2 million Australians were living with T2DM, with 188 newly diagnosed patients per day per 100 000 population.^16^ The prevalence of T2DM is estimated at nearly 7% of the world's population, or 7079 patients per 100 000 by 2030.^17^ T2DM has become a global burden for the healthcare system and causes mortality of over 1 million deaths per year. Therefore, T2DM holds a central position in this review. Plasma proteins are predominantly made up of albumin (60%), globulins (36%), fibrinogen (4%) and others (2%) (Figure 2).

## 2 METHODOLOGY

**FIGURE 3:**
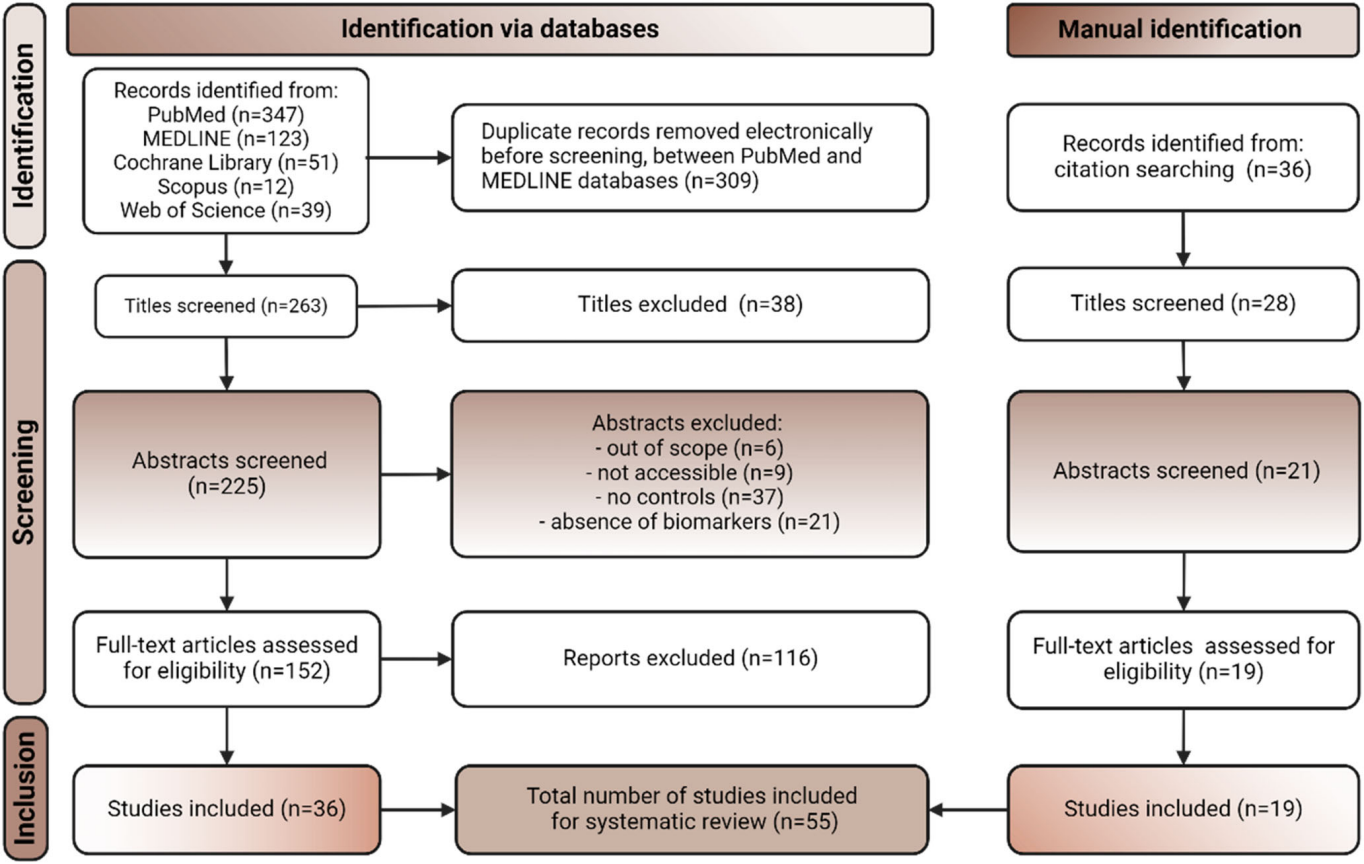
The PRISMA flowchart outlines the process for selecting the studies for the systematic review. It begins by identifying records from databases and other sources and then proceeds to screen these records to eliminate duplicates and irrelevant studies. Next, full‐text articles were assessed for eligibility and those that did not meet the criteria were excluded. Finally, the flowchart shows the number of studies included in the review (*n* = 55).

PubMed, MEDLINE, Cochrane Library, Scopus and Web of Science were used as a tool for searching articles published in English (Figure 3). Search terms were diabetes and blood parameters, diabetes and proteins, diabetes and inflammation and diabetes and low‐density lipoprotein (LDL)/high‐density lipoprotein (HDL). Filters applied were clinical data, guidelines, practical recommendations, research and reviews from the past 10 years (2013–2024) to ensure the inclusion of recent advancements, relevant data and improved methodologies. A comparative cross‐sectional review was conducted on 24 160 (10 055 T2DM patients and 14 105 CTL) study participants (aged 30–90 years). All participants with early‐stage diabetes (using insulin for at least 3 months) over a 15‐year follow‐up period were included in the review. Comorbidities such as hypertension and other diseases were included in the clinical reports. Conditions such as acute and chronic infections, stroke, trauma and hormonal disorders were excluded.

## 3 HAEMATOLOGICAL PROFILE OF T2DM SUBJECTS

WHO diagnostic criteria for diabetes (Table 1) are (1) fasting plasma glucose, (2) 2‐h post‐load plasma glucose after a 75 g oral glucose tolerance test (OGTT), (3) random blood glucose in the presence of signs and symptoms of diabetes and (4) glycosylated haemoglobin or glycated haemoglobin (HbA1c). HbA1c is an important diagnostic indicator of diabetes as it shows the average concentration of glucose in the past 3 months.^2^, ^19^ Table 2 shows the haematological alterations in patients with T2DM in white blood cells (WBC), including neutrophils, lymphocytes, monocytes, eosinophils and basophils, along with red blood cells (RBCs) and platelets. With every increase in 1000 cells/mm^3^ within the normal range of total peripheral WBC count (6620 ± 1480 cells/mm^3^), the risk for diabetes increases by 7.6%.^20^ The consistent increases from 1.1‐ to 1.2‐fold compared to non‐diabetic individuals were provided in the three studies shown in Table 2. These WBCs are the innate immune system against microorganisms. A high WBC count predicts increased inflammation and the development of T2DM.^21^ The number of total WBCs, neutrophils, lymphocytes, monocytes, eosinophils and basophils increased (Table 2).

**TABLE 1:** World Health Organization diagnostic criteria for diabetes.

	Diagnostic criteria	Diabetes mellitus	Pre‐diabetes
1	Fasting plasma glucose	≥7.0 mmol/L (126 mg/dL)	6.1–6.9 mmol/L
2	2‐h post‐load plasma glucose	≥11.1 mmol/L (200 mg/dL)	7.8–11 mmol/L
3	Random plasma glucose	≥11.1 mmol/L (200 mg/dL)	‐
4	HbA1c	≥6.5% (48 mmol/mol)	5.7%–6.4% or ≥ 10% increase in A1C

*Note*: The four recommended diagnostic tests for diabetes include (1) measuring fasting plasma glucose, (2) conducting a 2‐h post‐load plasma glucose test after a 75‐g oral glucose tolerance test (OGTT), (3) assessing random blood glucose levels when signs and symptoms of diabetes are present and (4) determining glycosylated haemoglobin (HbA1c) levels. The criteria were abstracted from WHO 2019.^18^

**TABLE 2:** Clinical haematological biomarkers in non‐diabetic individuals (controls; CTL) versus those with type 2 diabetes mellitus (T2DM).

T2DM duration	Clinical variables	CTL mean ± SD	T2DM mean ± SD	Ratio (T2DM/CTL)	*P*‐Value	Inflammatory responses
Follow‐up^6^	WBC (10^3^ cells/μL)	6.50 ± 1.34 (*n* = 134)	7.01 ± 1.74 (*n* = 134)*	+1.1	0.004	↑Increased inflammation
After 1–3 month treatment^7^	WBC (10^9^ cells/L)	5.00 ± 1.40 (*n* = 94)	5.80 ± 2.30 (*n* = 52)*	+1.2	<0.01	
At least 3‐month period^8^	WBC (10^9^ cells/L)	7.20 ± 1.70 (*n* = 121)	8.60 ± 1.90 (*n* = 135)*	+1.2	<0.001	
Follow‐up^6^	Neutrophil 10^3^cells/μL)	3.80 ± 0.96 (*n* = 134)	4.14 ± 1.51 (*n* = 134)*	+1.1	0.020	↑Increased inflammation
At least 3‐month period^8^	Neutrophil (10^9^ cells/L)	4.30 ± 1.40 (*n* = 121)	5.10 ± 1.50 (*n* = 135)*	+1.2	<0.001	
5‐year follow‐up^9^	Neutrophil (10^9^ cells/L)	3.11 ± 0.05 (*n* = 728)	3.33 ± 0.11 (*n* = 138)	+1.1	0.064	
Follow‐up^6^	Lymphocytes (10^3^ cells/μL)	1.86 ± 0.54 (*n* = 134)	2.07 ± 0.62 (*n* = 134)*	+1.1	<0.001	↑Increased inflammation
At least 3‐month period^8^	Lymphocyte (10^9^ cells/L)	2.30 ± 0.60 (*n* = 121)	2.70 ± 0.90 (*n* = 135)*	+1.2	<0.001	
5‐year follow‐up^9^	Lymphocyte (10^9^ cells/L)	1.65 ± 0.02 (*n* = 728)	1.81 ± 0.05 (*n* = 138)*	+1.1	<0.001	
Follow‐up^6^	Monocytes (10^3^ cells/μL)	0.50 (0.4–0.7) (*n* = 134)	0.60 (0.40–0.70) (*n* = 134)	+1.2	0.150	↑Increased inflammation
At least 3‐month period^8^	Monocytes (10^9^ cells/L)	0.50 ± 0.20 (*n* = 121)	0.60 ± 0.20 (*n* = 135)*	+1.2	0.009	
5‐year follow‐up^9^	Monocytes (10^9^ cells/L)	0.30 ± 0.01 (*n* = 728)	0.31 ± 0.01 (*n* = 138)	+1.0	0.318	
Follow‐up^6^	Eosinophil (10^3^ cells/μL)	0.10 (0.10–0.20) (*n* = 134)	0.20 (0.10–0.30) (*n* = 134)*	+2.0	<0.001	↑Increased inflammation
<5 years (55%) and ≥5 years (65%)^10^	Eosinophil (10^3^ cells/μL)	0.15 ± 0.10 (*n* = 120)	0.19 ± 0.24 (*n* = 120)	+1.3	0.130	
Follow‐up^11^	Eosinophil (%)	2.09 ± 1.55 (*n* = 320)	3.95 ± 2.79 (*n* = 403)*	+1.9	<0.001	
Follow‐up^12^	Basophil (10^9^ cells/L)	(*n* = 793)	(*n* = 1059)*	+2.2	0.001	↑Increased inflammation
<5 years (55%) and ≥5 years (65%)^10^	Basophil (10^9^ cells/L)	0.02 ± 0.02 (*n* = 120)	0.04 ± 0.03 (*n* = 120)*	+2.0	<0.001	
6‐month period^13^	Basophils (%)	0.32 ± 0.17 (*n* = 30)	0.54 ± 0.20 (*n* = 30)*	+1.7	0.039	
Follow‐up^6^	RBC (10^6^ cells/μL)	5.20 ± 0.50 (*n* = 134)	5.10 ± 0.45 (*n* = 134)	−0.9	0.100	↓Reduced oxygen
<5 years (55%) and ≥5 years (65%)^10^	RBC (10^12^ cells/L)	5.31 ± 0.4 (*n* = 120)	4.89 ± 0.90 (*n* = 120)*	−0.9	<0.001	
<5 years (60%), 5–10 years (27%), >10 years (16%)^14^	RBC (x10^6^/μL)	5.37 ± 0.10 (*n* = 39)	4.24 ± 1.69 (*n* = 103)*	−0.8	<0.05	
Follow‐up^6^	Hgb (g/dL)	16.20 ± 1.30 (*n* = 134)	15.70 ± 1.20 (*n* = 134)*	−0.9	0.007	↓Reduced oxygen affinity
At least 3‐month period^8^	Hgb (g/dL)	14.10 ± 1.50 (*n* = 121)	13.90 ± 1.40 (*n* = 135)	−0.9	0.429	
<5 years (55%) and ≥5 years (65%)^10^	Hgb (g/L)	15.39 ± 1.49 (*n* = 120)	13.65 ± 2.37 (*n* = 120)*	−0.9	<0.001	
Follow‐up^6^	Platelet (10^3^ cells/μL)	247.30 ± 43.10 (*n* = 134)	262.80 ± 57.20 (*n* = 134)*	+1.1	0.013	↑Increased inflammation
At least 3‐month period^8^	Platelet (10^3^ cells/μL)	258.90 ± 56.30 (*n* = 121)	265.80 ± 68.20 (*n* = 135)	+1.0	0.395	
<5 years (55%) and ≥5 years (65%)^10^	Platelet (10^9^ cells/L)	257.20 ± 61.82 (*n* = 120)	260.30 ± 103.70 (*n* = 120)	+1.0	0.776	

*Note*: Elevated parameters in T2DM patients are displayed, and decreased parameters are indicated in grey.

Abbreviations: RBC, red blood cells; WBC, white blood cells.

* Denotes a statistically significant difference.

**FIGURE 4:**
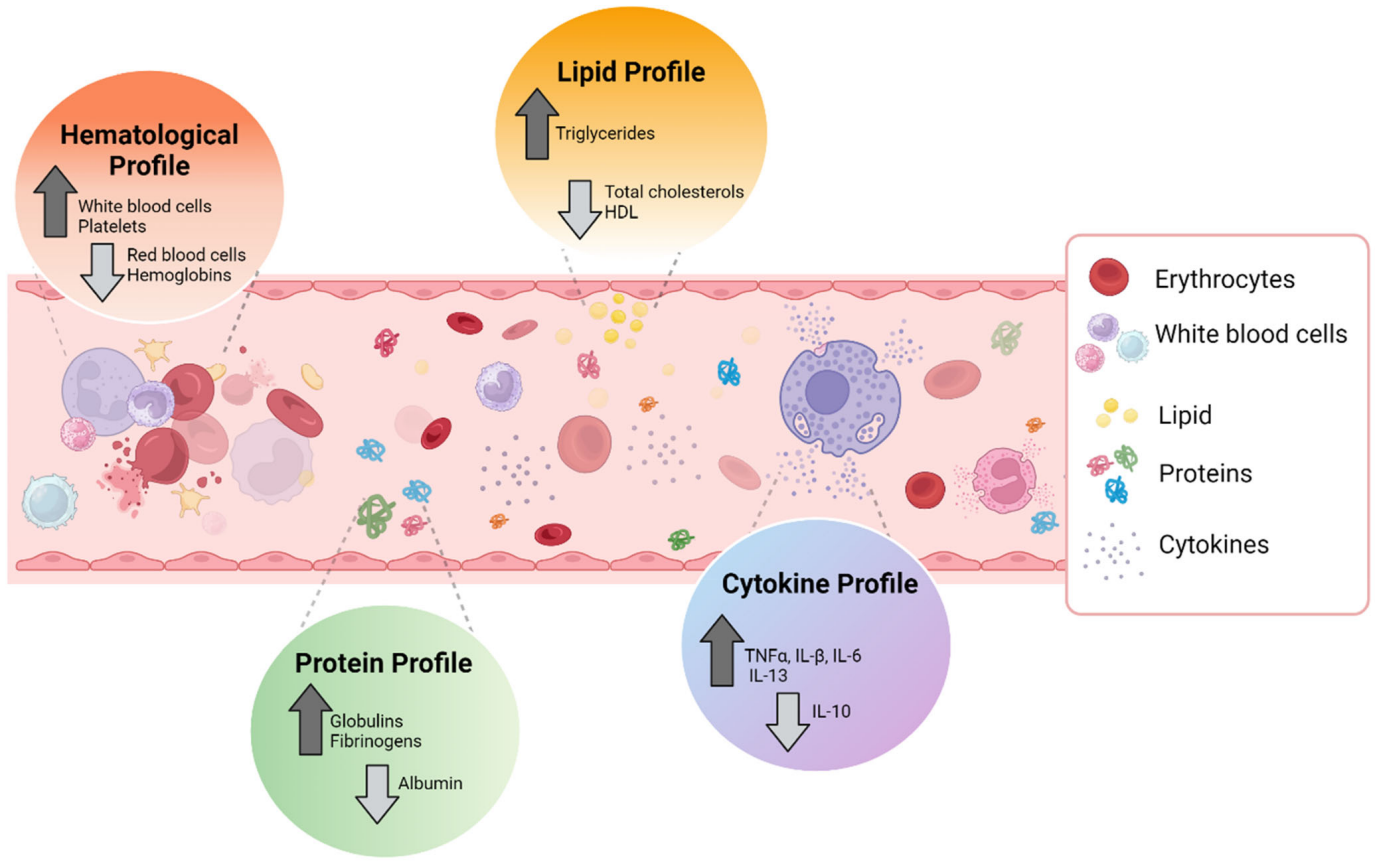
The four biomarker profiles in the blood components of T2DM subjects. The haematological profile shows an increased number of WBCs and platelets and lower RBC counts and haemoglobin. The protein profile presents an increase in the levels of globulin and fibrinogen, together with a reduction in albumin levels. The lipid profile reveals elevated levels of triglycerides along with reduced levels of total cholesterols and HDL. The cytokine profile exhibits an elevated expression of pro‐inflammatory cytokines (tumour necrosis factor alpha [TNF‐α], interleukin 1β [IL‐1β], IL‐6) coupled with an increased expression of anti‐inflammatory cytokine IL‐13 and a decreased expression of anti‐inflammatory cytokine IL‐10.

RBCs play a crucial role in transporting oxygen (O_2_) to all tissues in the body. As indicated in Table 2, patients with T2DM exhibited a 0.9‐fold reduction in the count of circulating RBCs and haemoglobin. This led to a decline in oxygen‐carrying capacity, which was associated with microvascular complications in Chinese individuals diagnosed with T2DM.^22^ Platelets play the primary role in haemostasis, thrombosis and wound healing. Increased mean platelet volume and platelet counts are commonly related to metabolic abnormalities such as diabetes, infection and inflammation. Elevated platelet counts in T2DM patients can lead to severe renal failure.^23^ They are also associated with increased levels of β‐thromboglobulin (β‐TG) and platelet factor 4 (PF4).^24^ The elevated levels of these two platelet markers cause platelet hyperactivation, which increases the risk of atherosclerosis in patients with T2DM. Patients with T2DM exhibit an increased platelet number due to glycation promotion in the two platelet proteins (β‐TG and PF4).^25^ In a broad context, those with T2DM exhibit changes in their blood composition. A notable increase of 1.1–1.2 times in WBCs and platelet counts among T2DM patients, compared to non‐diabetic counterparts, suggests the presence of inflammation. Conversely, a decrease in RBC count by 0.9 times signals impaired oxygen transport, which is linked to complications in diabetic patients (Figure 4).

## 4 T2DM PROTEIN PROFILE

Variations in blood plasma protein levels could serve as valuable predictors for forecasting the advancement and potential complications of T2DM. In addition, these protein levels have the potential to be employed as biomarkers to tailor personalized treatment approaches. The main components of blood plasma proteins are albumin (~60%), globulins (~36%) and fibrinogen (~4%). The proteomic profile of T2DM patients is summarized in Table 3, which includes a set of 13 highly abundant plasma proteins based on the dataset of high‐throughput studies. Distinct variances in plasma protein levels are evident in individuals with T2DM when compared to those without the condition. The 13 major differences are albumin, several liver‐produced acute‐phase proteins (α1‐globulin, α1‐acid glycoprotein [AGP], α1‐antitrypsin [AAT], C‐reactive protein (CRP); α2‐globulin, ceruloplasmin, haptoglobin; β‐globulin; γ‐globulin, IgA, IgM) and fibrinogen.

**TABLE 3:** Highly abundant proteins represented in non‐diabetic subjects (CTL) compared to type 2 diabetes mellitus (T2DM) patients (cases).

T2DM duration	Proteins	CTL mean ± SD	T2DM mean ± SD	Ratio T2DM/CTL	*p*‐Value	Inflammatory responses
>5 years^26^	Albumin (g/dL)	4.10 ± 0.42 (*n* = 30)	3.12 ± 0.58 (*n* = 30)*	−0.8	<0.05	↑Increased inflammation, chronic infections, impaired liver function, malnutrition
7 years^27^	Albumin (mg/g)	526 ± 37 (*n* = 50)	497 ± 55 (*n* = 55)*	−0.9	0.0311	
8.8–12 year follow‐up^28^	Albumin (g/dL)	4.20 ± 4.30 (*n* = 277)	3.87 ± 3.60 (*n* = 102)*	−0.9	<0.0001	
	Globulins					
9–11 years^29^	α1‐Globulin (g%)	0.16 ± 0.02, 41.1% (*n* = 21)	0.38 ± 0.06, 69.4% (*n* = 21)*	+2.3	<0.05	↑Acute‐phase reactant
9–11 years^29^	α1‐Globulin (g%)	0.29 ± 0.03, 43.3% (*n* = 20)	0.32 ± 0.07, 69.4% (*n* = 21)*	+1.1	<0.05	
8–10 years^30^	α1‐Globulin (g%)	0.16 ± 0.02 (*n* = 21)	0.38 ± 0.06 (*n* = 21)*	+2.4	<0.05	
7 years^27^	AGP (mg/g)	9.50 ± 2.90 (*n* = 50)	11.70 ± 2.90 (*n* = 55)*	+1.2	0.0011	↑Acute‐phase response
N/A^31^	AGP (mg/dL)	82.40 ± 28.90 (*n* = 33)	97.70 ± 28.00 (*n* = 89)*	+1.2	<0.05	
N/A^32^	AGP (mg/dL)	156.62 ± 12.39 (*n* = 29)	201.84 ± 10.70 (*n* = 30)*	+1.3	0.007	
7 years^27^	AAT (mg/g)	47.10 ± 15.30 (*n* = 50)	52.80 ± 11.80 (*n* = 55)	+1.1	0.061	↑Acute‐phase protein
Follow‐up^33^	AAT (mg/ml)	1.31 ± 0.23 (*n* = 158)	1.28 ± 0.28 (*n* = 163)	−1.0	>0.05	Lack of anti‐inflammation
Follow‐up^33^	AAT (<1 mg/mL)	*n* = 11	*n* = 23*	+2.1	<0.05	
7.2‐year follow‐up^34^	CRP (≥2.9 mg/L, %)	24.40 (*n* = 1951)	34.70 (*n* = 101)*	+1.4	0.02	↑Acute‐phase response
2 years^35^	CRP (mg/L)	Uncontrolled T2DM: 4.70 ± 2.80 (*n* = 25)	Uncontrolled T2DM: 91 ± 89.70 (*n* = 24)*	+19.4	<0.001	Promotes the binding of complement on bacteria
2 years^35^	CRP (mg/L)	With infection: 109.70 ± 108.60 (*n* = 6)	With infection: 118.70 ± 120.80 (*n* = 7)*	+1.1	<0.001	
9–11 years^29^	α2‐Globulin (g%)	0.77 ± 0.03, 19.0% (*n* = 21)	0.96 ± 0.05, 22.3% (*n* = 21)*	+1.2	<0.05	↑Acute‐phase response
1–3 month follow‐up^36^	α2‐Globulin (peptide)	244	433*	+1.8	<0.001	
8–10 years^30^	α2‐Globulin (g%)	0.77 ± 0.03 (*n* = 21)	0.96 ± 0.05 (*n* = 21)*	+1.3	<0.05	
7 years^27^	Ceruloplasmin (mg/g)	4.30 ± 1.60 (*n* = 50)	4.90 ± 1.30 (*n* = 55)	+1.1	0.079	↑Acute inflammation
N/A^37^	Ceruloplasmin (IU/L)	868.38 ± 198.80 (*n* = 90)	1222.82 ± 306.15 (*n* = 90)*	+1.4	<0.01	Copper transport, ferroxidase
3–21 years^38^	Ceruloplasmin (mg/L)	233.30 ± 37.80 (*n* = 594)	262.60 ± 40.90 (*n* = 49)*	+1.1	<0.001	
7 years^27^	Haptoglobin (mg/g)	12.70 ± 6.10 (*n* = 50)	18.20 ± 8.80 (*n* = 55)*	+1.4	<0.001	↑Acute inflammation
1–3 month follow‐up^36^	Haptoglobin (peptide)	3100	4214*	+1.4	<0.001	Hb binding, antibacterial effect
3–12 years^39^	Haptoglobin (ng/mL)	58.50 (3.7–465.4) (*n* = 269)	686.60 (79.1–8950) (*n* = 153)*	+11.7	<0.0001	
N/A^40^	β2‐Globulin (*𝜇*g/mL)	3.44 ± 1.12 (*n* = 30)	6.72 ± 2.032 (*n* = 30)*	+2.0	<0.001	↑ Increased chronic inflammation
1 year^41^	β2‐Globulin (𝜇g/mL)	0.10 (0.10–0.41) (*n* = 103)	0.41 (0.10–0.99) (*n* = 102)*	+4.1	0.002	
N/A^42^	β2‐Globulin (𝜇g/mL)	2.35 ± 0.56 (*n* = 21)	2.86 ± 0.95 (*n* = 21)*	+1.2	0.001	
9–11 years^29^	γ‐Globulin (g%)	1.48 ± 0.07, 20.4% (*n* = 21)	1.67 ± 0.09, 25.5% (*n* = 21)	+1.1	>0.05	↑Increased infection and inflammation
9–11 years^29^	γ‐Globulin (g%)	1.70 ± 0.10, 27.7% (*n* = 20)	1.80 ± 0.10, 22.6% (*n* = 21)*	+1.0	<0.05	Antigen‐specific binding
8–12 year follow‐up^43^	γ‐Globulin (g/100 mL)	1.16 (1.159–1.168) (*n* = 1861)	1.21 (1.203–1.212) (*n* = 442)*	+1.0	<0.05	
1–30 years^44^	IgA (mg/dL)	189 ± 65 (*n* = 132)	281 ± 119 (*n* = 169)*	+1.5	<0.01	↑Increased infection and inflammation
1–3 month^45^	IgA (mg/dL)	168 ± 88 x 10^−4^ (*n* = 24)	425 ± 223 x 10^−4^ (*n* = 24)*	+2.5	0.0001	
N/A^46^	IgA (mg/dL)	200 ± 74 (*n* = 30)	341 ± 182 (*n* = 66)*	+1.7	<0.005	
1–30 years^44^	IgG (g/L)	9.90 ± 1.85 (*n* = 132)	11.54 ± 2.39 (*n* = 169)*	+1.2	<0.01	↑Increased infection and inflammation
4–9 years^47^	IgG (g/L)	13.52 ± 1.11 (*n* = 35)	17.45 ± 3.61 (*n* = 35)*	+1.3	<0.05	
3–14 years^48^	IgG (g/L)	7.29 (4.68–11.65), (*n* = 78)	7.59 (6.43–8.35), (*n* = 80)*	+1.0	0.001	
≤1 year^49^	Fibrinogen (mg/dL)	324 ± 139 (*n* = 100)	656 ± 130 (*n* = 100)*	+2.0	<0.01	↑Acute inflammation
8–14 years^50^	Fibrinogen (mg/dL)	210 ± 12 (*n* = 7)	304 ± 31 (*n* = 6)*	+1.5	<0.05	Fibrin precursor.
N/A^51^	Fibrinogen (mg/dL)	314.40 ± 97.4 (*n* = 96)	386 ± 132.90 (*n* = 91)*	+1.2	<0.001	

*Note*: Increased concentrations in T2DM patients are displayed, and decreased levels in T2DM patients are shown in grey. N/A indicates not mentioned data. Abbreviations: AGP, α1‐acid glycoprotein; AAT, α1‐antitrypsin; CRP, C‐reactive protein; IgG, immunoglobin G; SD, standard deviation.

* Denotes a statistically significant difference.

Albumin synthesis is stimulated by insulin in the liver, and insulin deficiency is responsible for a reduction in albumin value. Insulin deficiency in T2DM patients leads to decreased levels of albumin (Table 3). A decrease of 10.8% serum albumin in 11 patients with T2DM compared to 45 CTL (odds ratio [OR] 0.36, 95% confidence interval [CI] 0.15–0.85, *p* = 0.019) showed that serum albumin might be a predictor for increased T2DM risk.^52^ Albumin plays a vital role in antioxidation and anti‐inflammation. Six commercial human albumin solutions were reported to inhibit the pro‐inflammatory cytokine TNF‐α.^53^ Serum albumin products decreased TNF‐α and IFN‐γ production by a mean of 37 ± 4.8% and 36 ± 9.6%, respectively.^53^ In addition, all other fractions (<3000 MW) of serum albumin significantly inhibited T‐lymphocyte production.^53^ In an 8.8‐year follow‐up study involving 379 subjects, albumin predicted T2DM (OR 0.75; 95% CI 0.58–0.96; *p* = 0.02) and was associated with heightened adipose tissue inflammation.^28^ Consequently, lower albumin levels may indicate an increased inflammatory state for patients with T2DM.

Hyperglycaemia induces glycation of circulating proteins such as albumin, fibrinogen, immunoglobin G (IgG), collagen and lipoproteins. These glycated plasma proteins lead to a series of deleterious effects in the body, including platelet activation, impaired fibrinolysis, increased reactive oxygen species (ROS) and impaired immune regulation. Glycation of albumin causes structural changes, leading to a loss of function and impaired glucose metabolism. These findings supported the strong link of glycated albumin (GA) with major diabetic complications such as microvascular conditions. Advanced glycation end products (AGEs) are compounds formed through a series of complex non‐enzymatic biochemical reactions. Diabetes is the primary factor in stimulating high levels of AGE formation.^54^ AGEs, such as GA, A1c, fructosamine and 1,5‐anhydroglucitol, are proteins and lipids that have undergone non‐enzymatic glycation reactions to become heterogeneous and irreversible compounds.^55^ Receptors for AGEs (RAGE) play a crucial role in the pathogenesis of diabetic complications.^56^ RAGE is a multiligand receptor that is found on macrophages, muscle cells and endothelial cells.^54^ The interaction of AGEs and RAGE on macrophages induces oxidant stress and activates nuclear factor‐κB (NF‐κB).^57^ NF‐κB promotes gene expression of pro‐inflammatory cytokines such as TNF‐α and interleukin 6 (IL‐6).^57^ Therefore, inhibition of AGE formation is considered in the prevention of diabetic complications.^54^

### 4.1 Acute‐Phase Proteins

The globulins comprise 36% of plasma proteins (Figure 2). There are four main globulin categories, including α1‐globulins, α2‐globulins, β‐globulins and γ‐globulins. Changes in globulin levels have also been associated with an increased risk of diabetes. For example, an increase in α1‐globulin is seen in the liver in response to acute inflammation resulting from acute‐phase reactants.^30^ In the group of α1‐globulins, there are three acute‐phase proteins: AGP, AAT and CRP overexpressed in T2DM. AGP is synthesized and secreted by hepatocytes, primarily serving as one of the most prominent acute‐phase proteins that can identify individuals who are at risk of T2DM.^58^ Another study investigated AGP levels that were significantly higher (1.3 times) in diabetic patients (2018.43 ± 107.83 μg/mL) without metabolic syndrome (*p* = 0.015) compared to healthy non‐diabetic subjects (1566.23 ± 120.39 μg/mL) (Table 3).^32^ Therefore, AGP has been used as a prediction for developing T2DM in adults.^59^

AAT is a major protease inhibitor produced in the liver and released into the blood. It is a member of the serpin family, which is the serine protease inhibitor. AAT was first identified as an inhibitor of proteinase trypsin. Anti‐inflammation is the primary function of AAT. In certain inflammatory conditions, the liver produces AAT as an acute‐phase protein to react with cytokines and chemokines. AAT deficiency (AATD) is associated with an elevated susceptibility to T2DM. In one study, the number of diabetic patients with low AAT levels (<1.0 mg/mL), together with the frequency of AATD genotypes, was 50% higher than CTL (*p* < 0.05).^33^ Other studies show that lower levels of plasma AAT were recorded in T2DM patients.^33^, ^60^ These findings showed that T2DM patients lack anti‐inflammation, a function of AAT. However, in another cohort, an increase in AAT concentration in T2DM patients (11.7 ± 2.9) was reported to be 1.1 times higher than in non‐diabetic participants (9.5 ± 2.9).^27^ This alarmed the high inflammatory state in these T2DM patients.

CRP is the major acute‐phase protein synthesized primarily by liver hepatocytes but also by smooth muscle cells, macrophages, endothelial cells, lymphocytes and adipocytes in response to inflammation. Also, CRP levels increase in cases of viral infections such as influenza and COVID‐19. Pro‐inflammatory cytokines, including TNF‐α and IL‐6, are produced by adipose tissue and subsequently regulate the hepatic synthesis of CRP in the liver. The role of these two cytokines was recorded in lipid and glucose metabolism in human adipose tissue. In people with diabetes, CRP levels are often elevated, indicating the presence of chronic low‐grade inflammation, which is a risk factor for cardiovascular disease. The multinational World Health Organization MONICA project performed a 7.2‐year follow‐up study with nearly 15 000 male and female participants (age 25–74 years) from 1984 to 1998. The result showed that men with CRP levels in the highest quartile (CRP ≥2.91 mg/L) exhibited a 2.7 times higher risk of developing diabetes.^34^ CRP was demonstrated as a predictor of inflammation and incident diabetes.^34^ Patients with T2DM had an increased level of CRP. A cross‐sectional study with 62 poorly controlled T2DM patients showed that 96.7% of T2DM individuals had elevated CRP levels (≥ 10 mg/L) in contrast to healthy CTL subjects (Table 3).^35^ The study also concluded that hyperglycaemia is a potential factor, contributing to increased CRP levels in patients with uncontrolled T2DM.

Ceruloplasmin is the major copper‐carrying protein. It transports approximately 95% copper in the blood and exhibits a ferroxidase activity that catalyses the oxidation of ferrous iron into ferric iron, assisting iron transportation of transferrin in the bloodstream. Ceruloplasmin is an acute‐phase reactant that is produced by hepatocytes when induced by cytokines released from activated monocytes and macrophages. The level of ceruloplasmin was significantly higher among T2DM patients as compared to CTL (1222.82 ± 306.15 IU/L vs. 868.38 ± 198.80 IU/L, *p* < 0.01) in diabetes.^37^, ^61^ Elevated ceruloplasmin levels have been reported as a marker of abnormally high oxidative stress in T2DM.^62^ Diabetic retinopathy is a common microvascular complication that can cause blindness. A study reported that high levels of ceruloplasmin are related to T2DM patients with retinopathy (*p* < 0.01) (Table 3).^37^ Increased ceruloplasmin blood levels (4.9 ± 1.3 mg/g) are also seen in other T2DM studies.^27^, ^36^, ^38^ IFN‐γ and TNF‐α are responsible for the induction of ceruloplasmin synthesis by human myeloid cells.

Haptoglobin binds to free haemoglobin and forms a high‐affinity complex that is eliminated from circulation by the reticulocyte system, CD163‐positive macrophages and hepatocytes. The complex's removal time is approximately 20 min.^63^ Haptoglobin acts as a clearance protein and antioxidant, therefore reducing the oxidative activity of heme iron. With antioxidant properties, haptoglobin is involved in innate immune mechanisms, restricts the accessibility of iron to pathogens and stimulates tissue repair in an inflammatory event. The expression of macrophage produces pro‐inflammatory cytokines (IL‐1, IL‐6, TNF‐α), insulin and bacterial endotoxin that induce the expression of haptoglobin. Levels of urinary haptoglobin increased 11‐fold in patients with T2DM compared to patients without T2DM in an Asian T2DM cohort.^39^ The urinary haptoglobin value can identify early‐stage diabetes and predict its progression irrespective of urinary albumin levels and conventional risk factors.

β‐Globulins comprise about 16% of serum proteins in the blood of healthy individuals. Elevated levels of β‐globulin can be seen in many conditions, such as infections, hypothyroidism, iron deficiency anaemia, chronic inflammation and T2DM. In contrast, β‐globulin deficiency is commonly caused by malnutrition and liver and kidney disease.^64^ A significantly high level of serum β2‐globulins (≥1.8 mg/L) was positively correlated with T2DM patients with diabetic nephropathy (*p* < 0.001).^64^, ^65^ Elevated serum and urinary elevated levels of β2‐globulins have a high prevalence in T2DM patients with multiple diabetic complications, such as retinopathy and nephropathy.^66^ An increased correlation between β2‐globulins and the development of end‐stage renal disease was observed in Indian individuals diagnosed with T2DM.^66^ Urinary and serum β2‐globulins were used as potential clinical biomarkers in the detection of early nephropathy in T2DM patients (Table 3).^41^, ^42^ Overall, β2‐globulins serve as predictors for patients with T2DM who have complications.^65^, ^67^

γ‐Globulins are the most abundant proteins after albumin. The most significant γ‐globulins are immunoglobins. The five main classes of immunoglobulins are IgG, IgM, IgA, IgD and IgE. γ‐Globulins have been used as a marker of the acute adaptive immune system and have a close association with infection, inflammation, obesity and T2DM. In a study involving 2530 participants, elevated levels of γ‐globulin were identified as a noteworthy predictor of T2DM, and γ‐globulin was linked to a 20% higher incidence of T2DM.^43^ Total γ‐globulin concentrations were associated with higher body mass index (BMI) and also increased in people with impaired glucose tolerance or T2DM.^43^, ^68^ Significant increases in concentrations of serum IgG (by 35.2%, *p* < 0.001) and IgA (by 82.7%, p < 0.001) were reported in 110 diabetic patients compared to 111 healthy subjects. Consistent with the study on 169 diabetic patients and 132 CTL, the increase in serum IgA and IgG concentrations was recorded (IgG concentration in diabetic patients 11.54 ± 2.39 g/L vs. 9.905 ± 1.85 g/L in CTL, *p* < 0.001).^44^ High IgA and IgG concentrations were observed in over 41% and 11.8% of diabetic patients, respectively.^44^ Changes in IgA levels can be monitored in assessing the inflammatory status of T2DM patients. Poor glycaemic control was associated with an increase in serum IgA and IgG levels (Table 3).

Fibrinogen is a plasma glycoprotein produced by the liver and is involved in haemostasis.^69^ It makes up approximately 4% of total plasma protein. Fibrinogen acts as an acute‐phase reactant, as its concentration significantly increases during inflammatory events.^70^ Hyperfibrinogenaemia is defined as plasma fibrinogen levels >310 mg/dL (reference range 210–310 mg/dL).^71^ Hyperfibrinogenaemia contributes to the development of pathological thrombosis. Elevated levels of fibrinogen have been clinically used as a risk marker of inflammation, cancer, diabetes and thrombotic events, such as myocardial infarction and strokes.^49^, ^72^ One study demonstrated that higher fibrinogen and WBC count, together with lower albumin, can predict T2DM.^59^ The plasma fibrinogen levels in 100 patients with T2DM (656 ± 130 mg/dL) were observed as significantly higher than 100 CTL (324 ± 139 mg/dL) (*p* < 0.01) (Table 3).^49^ Another study found a 1.5‐fold increase in fibrinogen production in T2DM patients compared to normal subjects (Table 3).^50^

In addition, increased levels of fibrinogen are significantly correlated with age, hypertension, BMI, smoking, ischaemic heart disease and HbA1c.^50^ In a study of 96 non‐diabetic CTL subjects and 91 patients with T2DM, elevated fibrinogen production was strongly associated with an increased concentration of glucagon.^73^ Another study highlighted a significant increase in plasma fibrinogen levels among the diabetic group in comparison to the non‐diabetic subset (386.0 ± 132.9 vs. 314.4 ± 97.4; *p* < 0.001) (Table 3).^51^ The authors also confirmed a positive correlation between HbA1c and fibrinogen levels (*r* = 0.49), showing that poorer glycaemic control is associated with higher fibrinogen levels. Hyperglycaemia in T2DM leads to hyperfibrinogenaemia by activating the coagulative pathway, inducing a cascade of clotting factors and promoting haemostasis. The activation of thrombin formation subsequently stimulates the synthesis of fibrinogen.^74^ Overall, patients with T2DM typically exhibit elevated levels of globulins and fibrinogen in their protein profile, coupled with a decrease in albumin levels (Figure 4).

## 5 T2DM CYTOKINE PROFILE

Cytokine analysis has been employed to elucidate the link between pro‐inflammatory and anti‐inflammatory cytokines in individuals with obesity and T2DM.^75^, ^76^ Understanding dysfunctional immune cell metabolism and changes in cytokine blood concentrations aids the diagnosis and development of interventions for patients suffering from T2DM. Inflammation is a central concern in the pathogenesis of T2DM. The hypertrophied adipocytes and M1 macrophages upregulate the secretion of pro‐inflammatory cytokines, whereas the expression of anti‐inflammatory cytokines is reduced.^76^, ^77^ Changes in cytokine patterns disrupt both innate and adaptive immunity, resulting in either acute or chronic inflammation in the context of obesity and diabetes. Pro‐inflammatory cytokines (TNF‐α, IL‐1β and IL‐6) and their anti‐inflammatory counterparts (IFN‐α, IL‐10 and IL‐13) have been summarized in this review due to the major alternatives and common use in clinical tests. TNF‐α is a pro‐inflammatory cytokine expressed by macrophages and monocytes during acute inflammation. It oversees a wide array of signalling events within cells, ultimately leading to necrosis or apoptosis.^78^ TNF‐α reduces insulin‐dependent glucose uptake by impeding serine autophosphorylation of insulin receptor substrate‐1, which inhibits circulating insulin, indicating an involvement of TNF‐α in insulin resistance.^79^, ^80^, ^81^ Overexpression of TNF‐α has a strong correlation with the pathogenesis of numerous chronic inflammatory diseases, such as obesity‐associated T2DM^82^ and rheumatoid arthritis.^78^ The increase in the amount of TNF‐α promotes insulin resistance in adipocytes and peripheral tissues, resulting in the development of T2DM; these were reported in two meta‐analyses (Table 4).^94^, ^95^ Overall, increased TNF‐α production in adipose tissue is strongly correlated with obesity‐related insulin resistance and chronic inflammation in T2DM.

**TABLE 4:** Changes in cytokine expression in T2DM subjects.

T2DM duration	Cytokines	CTL mean ± SD	T2DM mean ± SD	Ratio (T2DM/CTL)	*p*‐Value
**Pro‐inflammatory markers**					
≥3 months^83^ ≤1 year^75^ N/A^82^	TNF‐α (pg/mL) TNF‐α (pg/mL) TNF‐α (pg/mL)	86.30 (68.90–121.50), (*n* = 23) 46.30 ± 11 (*n* = 18) 6.19 ± 3.01 (*n* = 63)	161.70 (98.60–197.50), (*n* = 25)* 100.30 ± 9.60 (*n* = 19)* 7.51 ± 2.48 (*n* = 65)*	+1.9 +2.2 +1.2	0.012 0.005 0.008
≥3 months^83^ 2‐year follow up^84^ Newly diagnosed^85^	IL‐1β (pg/mL) IL‐1β (pg/mL) IL‐1β (pg/mL)	16.47 (11.40–30.30), (*n* = 23) 12.71 ± 9.10 (*n* = 20) 20.70 ± 1.30 (*n* = 52)	48.98 (33.80–76.60), (*n* = 25)* 100 ± 98.44 (*n* = 30)* 22.30 ± 1.50 (*n* = 58)	+3.0 +7.9 +1.1	0.00005 <0.001 >0.05
≥3 months^83^ N/A^86^ 4 years^87^	IL‐6 (pg/mL) IL‐6 (pg/mL) IL‐6 (pg/mL)	8.43 (0.44–21.18), (*n* = 23) 2.08 (1.50–4.20), (*n* = 304) 1.38 (0.91–2.05), (*n* = 362)	239.41 (31–1018), (*n* = 25)* 4.10 (2.30–5.60), (*n* = 63)* 2.00 (1.43–2.78), (*n* = 188)*	+4.7 +1.5 +1.5	0.00005 <0.0001 <0.001
**Anti‐inflammatory markers**					
≥3 months^83^	IFN‐α (pg/mL)	1.35 (0.74–1.63), (*n* = 23)	2.28 (1.94–2.49), (*n* = 25)*	+1.7	0.003
Newly diagnosed^88^	IFN‐α (IU/mL)	0.30 ± 0.70 (0–3), (*n* = 20)	11.60 ± 8.70 (0–33), (*n* = 31)*	+38.7	<0.0001
N/A^89^	IFN‐α (pg/mL)	26.20 (0–84), (*n* = 24)	0 (0–20), (*n* = 20)*	−26.2	0.012
≥3 months^83^	IL‐10 (pg/mL)	16.11 ± 2.27 (*n* = 120)	9.53 ± 2.27 (*n* = 131)*	−0.5	0.0002
N/A^90^	IL‐10 (pg/mL)	1402 (1331–1474), (*n* = 184)	377 (358–396), (*n* = 184)*	−0.3	<0.005
Newly diagnosed^91^	IL‐10 (pg/mL)	14.52 ± 9.71 (*n* = 35)	10.93 ± 8.62 (*n* = 35)*	−0.8	<0.015
≥3 months^83^	IL‐13 (pg/mL)	2.25 (0.665–7.03), (*n* = 23)	5.75 (2.56–10.01), (*n* = 25)*	+2.6	0.045
>5 years^92^	IL‐13 (pg/mL)	62.80 ± 10.70 (*n* = 115)	79.80 ± 41.80 (*n* = 115)*	+1.3	<0.05
11 years^93^	IL‐13 (pg/mL)	106 ± 133, (*n* = 10)	108 ± 111, (*n* = 11)	+1.0	0.801

*Note*: Increased cytokines and decreased (grey) cytokines in T2DM patients are shown. N/A indicates not mentioned data.

* Denotes statistically significant differences in the studies.

IL‐1, a pro‐inflammatory family cytokine, comprises IL‐1α, IL‐1β and IL‐1 receptor antagonist (IL‐1Ra). IL‐1β is an inflammation‐promoting cytokine secreted by islet β‐cell macrophages. Hyperglycaemia in T2DM induces β‐cell apoptosis, causing decreased β‐cell mass or loss of β‐cell. The destruction of pancreatic β‐cells promotes IL‐1β production.^96^ During inflammation, IL‐1β is strictly regulated by IL‐1Ra. Macrophages regulate and balance the ratio of IL‐1β/IL‐1Ra in response to infection or damage. IL‐1β inhibits insulin secretion and induces β‐cell apoptosis, leading to T2DM. Serum IL‐1β levels were significantly higher in T2DM patients than in CTL subjects (Table 4).^84^ Additionally, a positive correlation between IL‐1β levels and glycaemic control in T2DM was shown.^84^ This is consistent with the results of another study that exhibited elevated IL‐1β levels in T2DM.^85^ IL‐6 is a pro‐inflammatory cytokine produced by several immune cells (macrophages, dendritic cells, T and B cells) and other cells such as fibroblasts, endothelial cells and islet β‐cells in response to infections, trauma and cellular injury.^97^ IL‐6 plays an essential role in B‐cell differentiation, along with IL‐2 and IL‐10.^98^ It enhances a transcriptional inflammatory response through the IL‐6 receptor. Dysregulation of IL‐6 production is linked to the pathogenesis of T2DM, insulin resistance and inflammation. The increase in IL‐6 concentrations was used as a biomarker to predict the development of T2DM (Table 4).^83^, ^86^, ^87^

Long‐term overexpression of interferon alpha (IFN‐α) can be cytotoxic to both pancreatic islet cells and liver hepatocytes. Elevated IFN‐α levels increase IL‐1 concentration, which is cytotoxic to β‐cells.^99^ In patients with insulin‐dependent diabetes group, higher plasma IFN‐α levels were reported in 70% of patients and associated with coxsackievirus B infection than those in healthy CTL without any metabolic, immunological and infectious disease (T2DM cases: 11.6 ± 8.7 vs. CTL: 0.3 ± 0.7 IU/mL, *p* < 0.0001) (Table 4).^88^ These results are consistent with another study that performed a cytokine analysis, including anti‐inflammatory and pro‐inflammatory cytokines in the whole blood of T2DM patients with cardiovascular disease: 2.28 (1.94–2.49) versus CTL: 1.35 (0.74–1.63) pg/mL, *p* = 0.003.^83^ Remarkably, an increased expression of anti‐inflammatory cytokines IFN‐α and IL‐13 was observed in those T2DM patients, which may help mitigate the damage caused by an elevated pro‐inflammatory response.^83^ However, another study demonstrated decreased levels of IFN‐α in diabetic patients with and without retinopathy.^89^

IL‐10 is an anti‐inflammatory protein produced by T cells, B cells, monocytes and macrophages.^100^ IL‐10 levels were significantly lower in T2DM patients compared to CTL subjects (cases: 10.93 ± 8.62 vs. CTL: 14.52 ± 9.71 pg/mL, *p* < 0.015).^90^, ^91^, ^101^ These findings indicate that decreased production capacity of the anti‐inflammatory cytokine IL‐10 is linked with metabolic syndrome and can be a risk factor for T2DM.^90^, ^91^, ^101^ IL‐13 is classified as an anti‐inflammatory cytokine produced by activated T‐helper 2 cells.^102^ IL‐13 neutralizes pro‐inflammatory cytokines (such as TNF‐α and IL‐6), which were associated with the development of insulin resistance in T2DM. Adipose tissues or adipocytes are a source of IL‐13. IL‐13 enhances macrophage polarization towards the M2 phenotype and aids insulin regulation.^103^ M2 macrophages secrete anti‐inflammatory cytokines promoting cell proliferation and tissue repair, whereas M1 macrophage produces pro‐inflammatory cytokines causing tissue damage and inhibiting cell proliferation.^104^ Remarkably, a significantly increased expression of IL‐13 was recorded in a group of T2DM patients when compared to non‐diabetic subjects (cases: 5.75 [2.56–10.01] vs CTL: 2.25 [0.665–7.03] pg/mL, *p* = 0.045) (Table 4).^83^ This finding may be explained by the fact that the serum IL‐13 showed a strong positive correlation with insulin resistance.^83^, ^92^, ^93^ In addition, adipose tissues from lean subjects preferentially secrete anti‐inflammatory cytokines such as adiponectin, IL‐10, IL‐4 and IL‐13. Thus, an increased IL‐13 level was observed in T2DM patients.^105^ Analysis of the cytokines in T2DM patients revealed elevated levels of both pro‐inflammatory (TNF‐α, IL‐1β, IL‐6) and anti‐inflammatory cytokines (IFN‐α and IL‐13) accompanied by a reduction in the anti‐inflammatory cytokines IFN‐α and IL‐10 (Figure 4). The alterations result in an impaired immune system and metabolic imbalance that increase the susceptibility of T2DM patients to severe complications.

## 6 T2DM LIPID PROFILE

Lipids perform three main functions within the body: (1) structural components (triglycerides, phospholipids and sterols) of cell membranes, (2) energy storage in adipose cells (cholesterol esters and triglycerides) and (3) regulating and signalling (internal temperature and hormones).^106^ There are three primary forms of lipids: triglycerides (>95% of dietary lipids), phospholipids (2% of nutritional lipids) and sterols. Phospholipids, which make up about 2% of dietary lipids, are the main components of the bilayer membranes of cells, which protect the cells and transport nutrients and proteins passively and actively.^107^ High blood triglyceride levels (≥2.0 mmol/L) are prevalent in patients with poorly controlled diabetes, especially with macrovascular complications.^108^ Cholesterol is produced by hepatic cells in the liver and is essential for metabolic processes related to hormones. Lipoproteins are divided into four primary fractions based on their density: very‐low‐density (VLDL), intermediate‐density (IDL), low‐density (LDL) and high‐density lipoproteins (HDL) (Table 5). VLDL molecules are unable to penetrate the arterial tunica intima due to their larger size (diameter >50 nm). The smaller IDL (25–35 nm) contains apolipoprotein E (ApoE), triglycerides and cholesterol, formed by the breaking down of VLDL in muscles and adipose tissues. LDL (21–24 nm) is subsequently metabolized from IDL and transports cholesterol to tissues. HDL (8–12 nm), produced in the liver and intestine, are rich in cholesterol and transport cholesterols from tissues to the liver. HDL removes LDL from the bloodstream by transporting it to the liver, reducing the risk of cardiovascular disease.^110^

**TABLE 5:** Serum lipid levels in type 2 diabetes mellitus (T2DM) individuals.

T2DM duration	Cholesterol/Apo B	CTL mean ± SD	T2DM mean ± SD	Ratio (T2DM/CTL)	*p*‐Value
9–10 years^29^ 3–14 years^48^ 7 years^109^	Total cholesterol (mg/dL) Total cholesterol (mg/dL) Total cholesterol (mg/dL)	122.4 ± 7.2 (*n* = 21) 93.6 ± 19.8 (*n* = 78) 205 (177–228) (*n* = 803)	120.2 ± 1.1 (*n* = 21) 90 ± 25.2 (*n* = 80) 174 (152–198) (*n* = 611)*	−0.9 −0.9 −0.8	>0.05 0.184 <0.001
6‐month period^13^ Follow‐up^33^ 7 years^109^	HDL cholesterol (mg/dL) HDL cholesterol (mg/dL) HDL cholesterol (mg/dL)	35.8 ± 13.9 (*n* = 30) 23.8 ± 5.90 (*n* = 158) 48 (39–59) (*n* = 803)	21.6 ± 13.5 (*n* = 30)* 20.9 ± 6.1 (*n* = 163)* 45 (37–53) (*n* = 611)*	−0.6 −0.9 −0.9	<0.001 <0.001 <0.001
7 years^109^ 1 year^41^ 3–12 year^39^	LDL cholesterol (mg/dL) LDL cholesterol (mg/dL) LDL cholesterol (mg/dL)	126 (103–148) (*n* = 803) 1.4 (1.02–1.7) (*n* = 103) 50.4 ± 14.4 (*n* = 269)	97 (79–119) (*n* = 611)* 3.0 (1.4–3.9) (*n* = 102)* 54 ± 14.4 (*n* = 153)*	−0.8 +2.1 +1.1	<0.001 <0.001 0.023
2‐year follow‐up^84^ 7 years^109^ 1 year^41^	Triglycerides (mg/dL) Triglycerides (mg/dL) Triglycerides (mg/dL)	127.503 ± 31.808 (*n* = 30) 117 (87–159) (*n* = 803) 1.40 ± 0.81 (*n* = 103)	152.803 ± 17.385 (*n* = 30)* 134 (92–197) (*n* = 611) * 2.13 ± 0.93 (*n* = 102)*	+1.2 +1.1 +1.5	<0.001 <0.001 <0.001
7 years^109^ ≥1–3 month^7^	Apo B (mg/dL) Apo B (mg/dL)	98 (84–114) (*n* = 803) 110 (20) (*n* = 94)	82 (71–94) (*n* = 611)* 100 (30) (*n* = 52)	−0.8 −0.9	<0.001 >0.05

*Note*: Elevated parameters are shown; grey denotes reduced parameters in patients with T2DM. Abbreviation: SD, standard deviation.

* Denotes statistically significant differences (*p* < 0.05).

Apolipoproteins (Apo) are plasma proteins that bind to lipids to form lipoproteins, which primarily transport lipids in the blood, cerebrospinal fluid and lymph.^111^ There are several classes and subclasses of apolipoproteins, including Apo A (A1‐5), Apo B (B48 and B100), Apo C (C1‐4), apo D, E, F, H, L, M and apo(a). ApoB is the primary apolipoprotein that carries chylomicrons, VLDL, IDL, LDL cholesterol and apo(a). ApoB has been clinically used to assess cardiovascular risk in T2DM patients.^109^ A decreased level of ApoB was recorded in a group of 611 patients with T2DM compared to the healthy group (98 mg/dL for non‐diabetic subjects vs. 82 mg/dL for T2DM patients, *p* < 0.001) (Table 5).^109^ ApoB level was a stronger predictor of coronary artery disease (CAD) than LDL cholesterol, especially in insulin resistance and T2DM patients.^109^ T2DM and insulin resistance are risk factors for CAD.^112^, ^113^ This equivalence is reflected in several factors, including low HDL cholesterol levels, increased small, dense LDL particles (>100 mg/dL) and elevated triglyceride levels (>150 mg/dL) (Figure 4).^84^, ^112^, ^113^, ^114^ However, the blood lipid concentration of those with T2DM shows a decrease in LDL cholesterol due to insulin resistance, which depletes LDL production.^115^ There are two potential mechanisms to explain the varied LDL levels in T2DM. First, insulin stimulates glucose uptake and triglyceride synthesis. An increased amount of glucose delivered to the liver triggers the liver to secrete larger VLDL particles and increases the numbers of IDL and LDL. Consequently, excessive LDL cholesterol is present in the blood, which leads to the deposition of plaque in the artery walls.^115^, ^116^ Second, diminished lipoprotein lipase activity is linked to a decline in triglyceride clearance. Lipoprotein lipase, a hydrolytic enzyme produced by tissues like muscle and adipose tissue, is predominantly regulated by insulin for the removal of triglycerides from circulation.^117^

## 7 STRENGTH, SCOPE OF REVIEW AND FUTURE PERSPECTIVE

The systematic analysis provides valuable data for clinical management and research, particularly for developing effective personalized biomaterials and prognostic tools to improve diabetic outcomes. However, there are several limitations to consider. Comparing biomarker data between control (non‐diabetic) and T2DM groups was challenging due to the limited number of studies, which resulted in data heterogeneity. We also recommend standardizing methodologies and reporting practices across studies to enhance comparability and reduce variability. Additionally, variables such as sex, residence, educational status, smoking, alcohol consumption, hypertension, infections, stroke, trauma, hormonal disorders and medication use were not included in this review. It is also important to note that the biomarkers discussed may not be specific indicators and can be influenced by various factors such as medication. Our goal was to explore their role in the broader context of T2DM. Poorly controlled T2DM can lead to complications and severe inflammation. Further research is needed to examine the relationship between these biomarkers and the clinical outcomes in T2DM patients to help reduce associated complications. Additionally, future studies could investigate more biomarkers to provide a comprehensive understanding of T2DM and explore the potential for developing a biomarker panel based on the findings presented.

## 8 CONCLUSION

This review presents the relationship between consistent changes in four profiles and inflammatory responses. The haematological profile of T2DM patients exhibits a notable increase in WBCs and platelet counts, suggesting the presence of inflammation. In turn, a decrease in RBC count impaired oxygen transport, which is linked to complications in diabetic patients. A positive correlation between T2DM and inflammatory events is confirmed by elevated levels of globulins and fibrinogen coupled with a decrease in albumin levels in the protein profile. An overexpression of pro‐inflammatory cytokines and a reduction in anti‐inflammatory cytokines are associated with increased risks of infections and metabolic abnormalities in T2DM patients. A significant decrease in total cholesterol and HDL, coupled with increased levels of triglycerides, is shown in the lipid profile of T2DM patients. However, the impact on LDL levels can vary, showing either increases or decreases, reflecting the influence of insulin resistance in T2DM. Analysing these biomarkers can offer a more comprehensive understanding of the pathophysiology of T2DM and help predict disease progression.

## AUTHOR CONTRIBUTIONS

Study concept and design: Thien Ngoc Le. Manuscript writing, drafting, revision and discussion: Thien Ngoc Le, Richard Bright and Krasimir Vasilev. Practical advice, revision, discussion and review: Richard Bright, Vi‐Khanh Truong, Jordan Li and Rajiv Juneja. Critical revision of the manuscript for important intellectual content: Richard Bright and Krasimir Vasilev.

## FUNDING INFORMATION

Krasimir Vasilev thanks NHMRC for grant GNT1194466.Richard Bright also thanks the Flinders Foundation for funding.

## CONFLICT OF INTEREST STATEMENT

The authors declare that they have no relationships or activities that might bias or be perceived to bias their work.

### PEER REVIEW

The peer review history for this article is available at https://www.webofscience.com/api/gateway/wos/peer‐review/10.1111/dom.15991.

## Data Availability

The data that support the findings of this study are available on request from the corresponding author. The data are not publicly available due to privacy or ethical restrictions.
